# Defective Function of Inhibitor of κB Kinase Subunit Beta Associated With Multiple Immune‐Mediated Disorders

**DOI:** 10.1111/exd.70206

**Published:** 2026-01-14

**Authors:** Kiril Malovitski, Noy Keller Rosenthal, Lubna Khair, David Hagin, Tal Freund, Eylon Sharoni, Alon Peled, Yarden Feller, Rawaa Ishtewy, Janan Mohamad, Ofer Sarig, Liat Samuelov, Eli Sprecher, Mor Pavlovsky

**Affiliations:** ^1^ Division of Dermatology Tel Aviv Sourasky Medical Center Tel Aviv Israel; ^2^ Gray Faculty of Medicine and Health Sciences Tel Aviv University Tel Aviv Israel; ^3^ Division of Internal Medicine, Allergy and Clinical Immunology Tel Aviv Sourasky Medical Center Tel Aviv Israel

**Keywords:** *IKBKB*, *NF‐κB*, vitiligo

## Abstract

Abnormal NF‐κB activity has been previously implicated in a range of immune‐mediated disorders. Here, we aimed to elucidate the genetic basis underlying the co‐occurrence of vitiligo, Addison's disease and granuloma annulare in a 43‐year‐old woman. Whole‐exome sequencing identified a heterozygous splice‐site variant (c.1364+1G>A, p.Met455fsTer1) in *IKBKB*, encoding the Inhibitor of κB kinase subunit beta (IKKβ), predicted to result in a premature stop codon. Immunoblotting of keratinocytes transfected with the mutant construct demonstrated the presence of a truncated form of IKKβ. Using a luciferase reporter assay under the control of NF‐κB–responsive element, we demonstrated significantly reduced activity of the mutant protein compared to wild‐type, supporting a loss‐of‐function mechanism. In line with this observation, the mutant protein was found to result in decreased expression levels of genes known to be regulated by NF‐κB. Furthermore, HeLa cells transfected with the p.Met455fsTer1 variant or *IKBKB*‐targeted siRNA exhibited markedly reduced levels of p105 and its processed form p50, compared with HeLa cells transfected with wild‐type *IKBKB* or control siRNA, respectively. Collectively, these findings indicate that a loss‐of‐function effect in *IKBKB* may underlie the co‐occurrence of a number of immune‐mediated disorders through disruption of NF‐κB signalling.

## 1 Background

The nuclear factor kappa‐light‐chain‐enhancer of activated B cells (NF‐κB) signalling pathway plays a central role in immune regulation, inflammation and cellular stress responses [1, 2, 3, 4, 5, 6, 7]. Heterozygous loss‐of‐function pathogenic variants in *NFKB1* are the most common monogenic cause of common variable immunodeficiency (CVID), presenting with hypogammaglobulinemia, recurrent respiratory infections and reduced switched memory B cells [8, 9, 10]. These patients frequently exhibit immune dysregulation, including autoimmunity [4, 11]. The IκB kinase β (IKKβ), encoded by the *IKBKB* gene, is a key catalytic component of the IKK complex [12]. Bi‐allelic loss‐of‐function variants in *IKBKB* lead to severe combined immunodeficiency [13, 14], whereas heterozygous gain‐of‐function variants in the same gene have been linked to autoimmune and autoinflammatory diseases [15, 16, 17]. Here, we identified a heterozygous loss‐of‐function variant in *IKBKB* resulting in the co‐occurrence of a number of immune‐mediated disorders in a single patient.

## 2 Experimental Design

### 2.1 Patient

The participant provided written informed consent according to a protocol approved by the Tel Aviv Medical Center institutional review board and the Israeli National Committee for Genetic Studies in adherence to the Helsinki principles. The clinical photographs were obtained following the patient's informed consent and her approval for publication.

### 2.2 Whole Exome Sequencing

Genomic DNA was extracted from peripheral blood leukocytes using the Gentra Puregene Blood Kit (Qiagen, Hilden, Germany) according to the manufacturer's instructions. Exome sequencing of the patient was performed at the Tel Aviv Sourasky Genomic Center as previously described [18]. All exome sequencing data were analysed using the Franklin platform by Genoox (http://www.Franklin.genoox.com). The NGS pipeline used is based on the BWA aligner [19] and the two variant callers: GATK HaplotypeCaller [20] and FreeBayes [21]. Rare variants were filtered using data from dbSNP155, the 1000 Genomes Project, HGMD, gnomAD, Ensembl, Exome Variant Server and an in‐house database of individual exomes. Variants were classified by predicted protein and splicing effects using PolyPhen‐2 [22], SIFT [23], Provean [24], ConSeq [25], Varsome [26], CADD [27] and SpliceAI [28].

### 2.3 Direct Sanger Sequencing

Genomic DNA was PCR‐amplified using oligonucleotide primer pairs spanning the variants of interest (Table S1) with Taq polymerase (Qiagen) as previously described [29]. Gel‐purified amplicons were subjected to bidirectional DNA sequencing and analysed as previously described [29].

### 2.4 Quantitative RT‐PCR

For quantitative real‐time PCR, cDNA was synthesised from 1000 ng of total RNA using qScript kit (Quanta Biosciences, Gaithersburg, MD, USA). cDNA PCR amplification with gene‐specific intron‐crossing oligonucleotides (Table S2) was performed as previously described [30]. Results were normalised to *GAPDH* mRNA levels.

### 2.5 Cell Cultures and Reagents

Primary keratinocytes (KCs) were isolated from adult skin obtained from plastic surgery specimens after having received written informed consent from the donors according to a protocol reviewed and approved by the Tel Aviv Medical Center institutional review board as previously described [31]. Primary KCs were maintained in KC growth medium (Lonza, Walkersville, MD, USA).

HeLa cells were cultured in high‐glucose Dulbecco modified Eagle medium with 10% FCS, 1% L‐glutamine and 1% penicillin/streptomycin (Biological Industries, Beit‐Haemek, Israel).

### 2.6 Expression Vectors

Human IKBKB cDNA constructs harbouring wildtype, as well as mutant (c.1364 + 1), sequences were cloned into pCDNA3.1 and labelled with DDDDK and V5 or DDDDK and 6xHis tags, respectively (Epoch Life Science, Missouri City, TX, USA). For overexpression studies, IKBKB constructs were transiently transfected into human KCs or HeLa cells grown to 80% confluence using Lipofectamine 3000 (Invitrogen, Waltham, MA, USA).

### 2.7 Gene Silencing

Human KCs or HeLa cells were cultured at 37°C in 5% CO_2_ in a humidified incubator. To downregulate IKBKB expression, we used human IKBKB‐specific small interfering RNA (siRNA) (sc‐35 644; Santa Cruz Biotechnology, Dallas, TX, USA). As a control, we used Stealth RNAi siRNA Negative Control Lo GC (Invitrogen). KCs and HeLa cells were transfected using 25 pmol siRNA, using Lipofectamine RNAiMax (Invitrogen).

### 2.8 Western Blotting

Protein extraction, electrophoresis, transfer onto a PVDF or a nitrocellulose membrane and blocking were performed as previously described [18]. Blots were incubated overnight at 4°C with a primary rabbit polyclonal anti‐DDDDK antibody or a primary rabbit monoclonal anti‐NF‐κB1 p105/p50 antibody (Table S3). Incubation with a secondary antibody and membrane development were performed as previously described [18]. To control for protein loading, we re‐probed the blots using a primary mouse monoclonal anti‐β‐actin antibody (Table S3). Protein levels were quantified using ImageJ software (National Institutes of Health, Bethesda, MD).

### 2.9 NF‐κB Reporter Assay

Human primary KCs generated as previously described [32] cultured in white flat‐bottom 96‐well microplates (Greiner Bio‐One GmbH) were co‐transfected using Lipofectamine 3000 transfection reagent with a luciferase reporter under an NF‐κB–responsive element, a Renilla expression vector and [1] *IKBKB‐*specific or control siRNA; [2] wild‐type *IKBKB* construct or an *IKBKB* construct harbouring the p.Met455fsTer1 variant. Twenty‐four hours after transfection, luciferase activity was measured using a dual luciferase assay (Promega). Readings were normalised to Renilla activity.

## 3 Statistical Analysis

Comparisons of values between two groups were performed by the unpaired or paired Student's *t*‐test. When more than two groups were evaluated, one‐way analysis of variance (ANOVA) with post hoc Tukey honestly significant difference (HSD) was performed. A value of *p* < 0.05 was considered statistically significant.

## 4 Questions Addressed

This study aimed to elucidate the underlying genetic aetiology in an individual presenting with a complex autoimmune phenotype including Addison's disease, vitiligo and granuloma annulare. Given the co‐occurrence of multiple autoimmune and autoinflammatory conditions, a monogenic or shared immune‐genetic basis was hypothesized.

## 5 Results

### 5.1 Clinical Features

**FIGURE 1:**
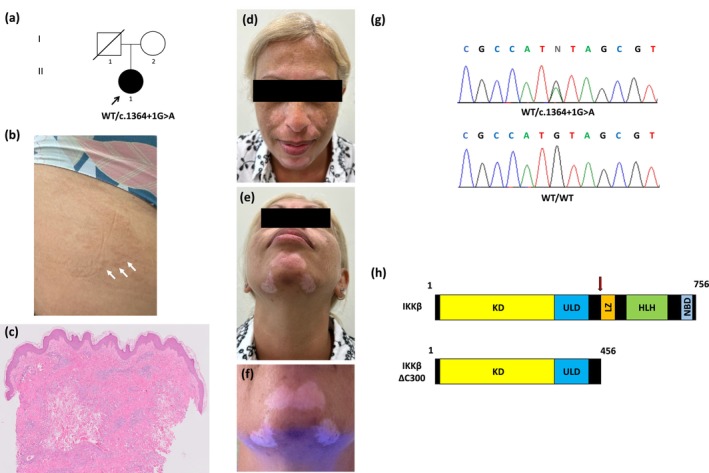
Pedigree, clinical features and variant analysis. (a) Pedigree. Black symbols denote affected individuals, based on examination and family history; (b, c) Annular erythematous plaque on the proximal thigh, showing a diffuse interstitial infiltrate predominantly composed of histiocytes and mild necrobiosis on histologic examination, consistent with granuloma annulare; (d–e) Hyperpigmentation due to Addison's disease and depigmented vitiligo patches; (f) Well‐demarcated white patches on the face, accentuated under Wood's lamp examination consistent with vitiligo; (g) Direct sequencing of IKBKB (NM_001556.3) revealed a heterozygous G>A transition (arrow) at position c.1364+1 of the cDNA sequence in individual II‐1 (upper panel); the wild‐type sequences (WT/WT) are given for comparison (lower panel); (h) Schematic representation of the wildtype IKKβ protein with its domains (upper panel) and of the truncated protein predicted to result from the IKBKB variant (lower panel).

A 43‐year‐old female of Caucasian origin with a known history of Addison's disease initially presented with an annular rash involving the dorsal aspect of the hands and thighs (Figure 1a,b). Histopathological examination revealed a diffuse interstitial infiltrate composed predominantly of histiocytes with slight necrobiosis, consistent with granuloma annulare (Figure 1c). Several months later, she developed depigmented patches over the face and dorsal hands (Figure 1d,e). Wood's lamp examination revealed bright white fluorescence within involved regions, consistent with vitiligo (Figure 1f). Importantly, there was no history of immunodeficiency, and immune cell phenotyping revealed no abnormalities (Figure S1a–c). The coexistence of three autoimmune and autoinflammatory manifestations raised the possibility of a common genetic aetiology.

### 5.2 Variant Analysis

Whole‐exome sequencing revealed a heterozygous c.1364+1G>A variant in *IKBKB* (NM_001556.3) (Figure 1g). Bioinformatic tools predicted the variant to result in donor splice site loss, and a premature termination codon which would lead to the translation of a truncated protein, p.Met455fsTer1, lacking several domains, including the NF‐κB essential modulator (NEMO)‐binding domain (Figure 1h). This domain was reported to be essential for the formation of a heterotrimeric IKKα‐IKKβ‐NEMO holocomplex [33]. The variant is absent from gnomAD and ClinVar databases. To confirm in vivo expression of the aberrant transcript, we performed direct sequencing of cDNA generated from patient‐derived cells, which revealed its presence at reduced levels (Figure S2).

### 5.3 Functional Consequences of the IKBKB Variant

**FIGURE 2:**
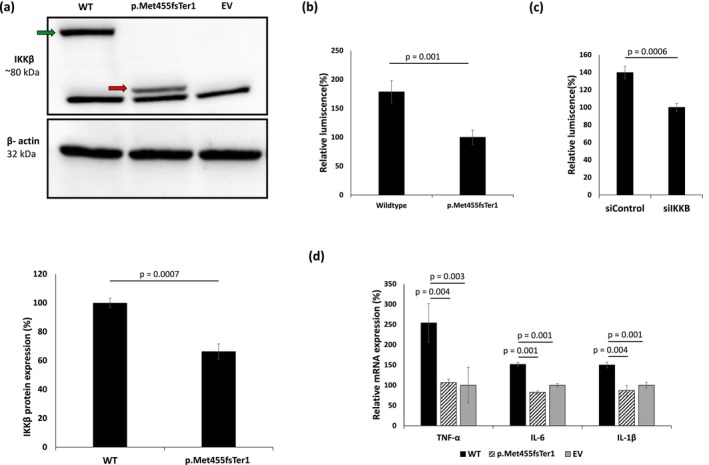
Consequences of the p.Met455fsTer1 IKBKB variant. (a) Primary KCs were transfected with expression constructs harbouring either *IKBKB* wild‐type sequence (WT) or the p.Met455fsTer1 variant. As a control, KCs were transfected with an empty vector (EV). Exogenous IKKβ expression was ascertained using immunoblotting with anti‐FLAG antibody. The green arrow denotes the full‐length protein and the red arrow denotes the truncated protein. β‐actin immunoblotting served as a protein loading control (upper panel). IKKβ protein level was quantified by ImageJ (lower panel). Results represent the mean ± SE of three independent experiments (*p* values were calculated using one‐way ANOVA and Tukey HSD test); (b) Primary KCs were co‐transfected with a NF‐κB–responsive luciferase reporter and with constructs harbouring either the wildtype (WT) *IKBKB* or the p.Met455fsTer1 variant. Luciferase activity was measured 24 h after co‐transfection and results were normalised to Renilla luciferase activity. Results represent mean ± SE of 3 independent experiments (*p* values were calculated using two‐sided *t*‐test); (c) Primary KCs were co‐transfected with a NF‐κB–responsive luciferase reporter and with either *IKBKB*‐specific siRNA (siIKBKB) or scramble siRNA (siControl). Luciferase activity was measured 24 h after co‐transfection, and results were normalised to Renilla luciferase activity. Results represent mean ± SE of 3 independent experiments (*p* values were calculated using two‐sided *t*‐test); (d) TNF, IL6 and IL1B mRNA levels were quantified using RT‐qPCR in primary human KCs transfected with expression vectors harbouring the wildtype (WT) *IKBKB* cDNA sequence or the p.Met455fsTer1 variant. As a control, KCs were transfected with an empty vector (EV). Results were normalised to *GAPDH* mRNA, represent the mean ± SE of three experiments and are expressed as a percentage of TNF, IL6 and IL1B mRNA expression in cells transfected with an empty vector (EV) (*p* values were calculated using one‐way ANOVA with post hoc Tukey HSD test).

To assess the functional consequence of the variant identified, we transfected primary human KCs with expression vectors harbouring either the wild‐type or the mutant *IKBKB*. Immunoblotting demonstrated that the pathogenic variant led to a significantly reduced expression of the truncated protein, despite comparable mRNA expression (Figure 2a; Figure S3). Additionally, immunofluorescent staining revealed that the truncated protein formed aggregates in the peri‐nuclear region, suggesting accumulation in the endoplasmic reticulum (Figure S4). To assess the effect of the pathogenic variant on NF‐κB signalling pathway activation, we used a luciferase reporter under NF‐κB–responsive elements, as previously reported [34]. Both KCs expressing the pathogenic *IKBKB* variant and those transfected with an *IKBKB*‐specific siRNA displayed significantly lower luciferase activity as compared with KCs transfected with wild‐type cDNA or control siRNA, respectively (Figure 2b,c; Figure S5). Furthermore, KCs transfected with the pathogenic *IKBKB* variant showed significantly reduced expression of several NF‐κB‐regulated cytokine genes, including *TNF*, *IL6* and *IL1B* (Figure 2d). Taken together, these results suggest a loss‐of‐function effect of the truncated protein.

### 5.4 The p.Met455fsTer1 Variant in IKBKB Affects NFKB1 Protein Expression

**FIGURE 3:**
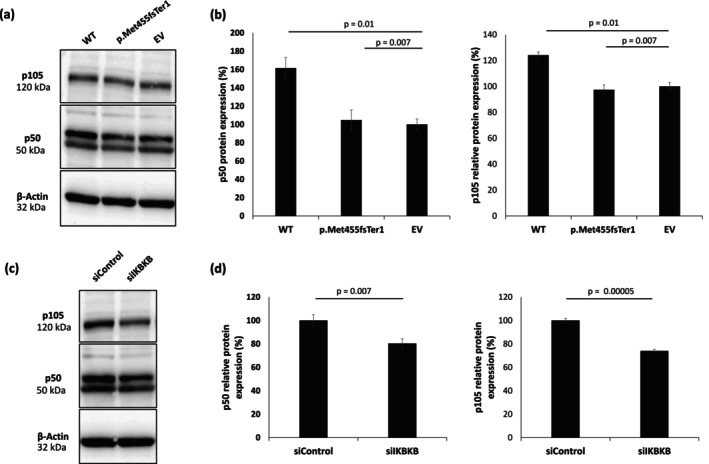
Effect of the p.Met455fsTer1 IKBKB variant on NFKB1 expression. (a) HeLa cells were transfected with expression constructs harbouring either *IKBKB* wild‐type sequence (WT) or the p.Met455fsTer1 variant. As a control, KCs were transfected with an empty vector (EV). p105 (upper panel) and p50 (middle panel) expression was ascertained using immunoblotting with anti‐NF‐κB1 p105/p50 antibody. β‐actin immunoblotting served as a protein loading control (lower panel); (b) p50 protein level were quantified by ImageJ (left panel). Results represent the mean ± SE of three independent experiments (*p* values were calculated using one‐way Anova and Tukey HSD test); p105 protein level was quantified by ImageJ (right panel). Results represent the mean ± SE of three independent experiments (*p* values were calculated using one‐way Anova and Tukey HSD test); (c) p105 (upper panel) and p50 (middle panel) were assessed in HeLa cells transfected with *IKBKB*‐specific siRNA (siIKBKB) or scramble siRNA (siControl) using immunoblotting with anti‐NF‐κB1 p105/p50 antibody. Results were normalised to β‐actin expression (bottom panel); (d) p50 (left) and p105 (right) expression levels were quantified by ImageJ and normalised to levels observed in HeLa transfected with control siRNA. Results represent the mean of three independent experiments (*p* values were calculated using two‐sided *t*‐test).

Next, we aimed at investigating the expression of the p50 DNA binding subunit and its precursor p105 encoded by the *NFKB1* gene in HeLa cells following *IKBKB* downregulation or transfection of the mutant *IKBKB* variant. HeLa cells transfected with the p.Met455fsTer1 variant or an *IKBKB*‐specific siRNA showed significantly reduced p50 and p105 expression, compared with cells transfected with the wild‐type construct or control siRNA, respectively (Figure 3). Notably, peripheral blood mononuclear cells (PBMCs) isolated from the patient exhibited impaired activation of the NF‐κB signalling pathway, as evidenced by reduced IκBα degradation and diminished phosphorylation of P65 following 15 min PMA and Ionomycin stimulation (Figure S6a,b). Taken collectively, these data suggest that the p.Met455fsTer1 variant may induce the observed autoimmune phenotype by affecting the activity of the NF‐κB signalling pathway, specifically by affecting p50 and p105 expression.

## 6 Conclusions and Perspectives

In this study, we describe a novel heterozygous splice‐site variant (c. 1364 + 1G>A, p.Met455fsTer1) in *IKBKB*, identified in a patient presenting with multiple autoimmune and autoinflammatory conditions, including Addison's disease, granuloma annulare and vitiligo. Direct sequencing of cDNA from patient‐derived cells demonstrated that the aberrant transcript is expressed in vivo, albeit at lower levels than the wild‐type transcript. This reduced expression suggests that the mutant mRNA may be subject to degradation through nonsense‐mediated decay (NMD) (Figure S2). Functional characterisation of this variant in primary human KCs revealed impaired NF‐κB signalling, evidenced by reduced IKKβ expression, perinuclear protein aggregation, diminished NF‐κB‐dependent transcriptional activity and reduced expression of downstream cytokines. In addition, this variant was associated with decreased expression of the *NFKB1*‐encoded p50 subunit, suggesting disruption of the canonical NF‐κB pathway. Furthermore, the splice‐derived truncated protein lacks the C‐terminal NEMO‐binding domain that is essential for IKK complex assembly and canonical NF‐κB activation, further supporting a loss‐of‐function mechanism [33].

NF‐κB is a transcription factor which regulates genes involved in immune responses, inflammation and cell survival [35]. Its activation is tightly controlled by the IKK complex, in which IKKβ, encoded by *IKBKB*, plays a central role in phosphorylating IκB inhibitors to enable NF‐κB nuclear translocation [36].

Notably, while bi allelic *IKBKB* loss‐of‐function variants have been associated with profound immunodeficiency and disrupted lymphocyte development [13, 37], our patient's immunophenotype was largely preserved with normal T‐ and B‐cell maturation and intact regulatory T‐cell populations. However, despite the normal immune cell composition, flow cytometry revealed impaired NF‐κB activation in patient‐derived PBMCs, evidenced by reduced p65 phosphorylation and impaired IκBα degradation following stimulation.

Our study has several limitations. Previous reports of biallelic IKBKB variants did not describe autoimmune or autoinflammatory manifestations in heterozygous carriers, which may reflect under recognition due to the severity of the biallelic phenotype or unique effects of our variant. In addition, our findings are based on a single family presenting with a novel phenotype, which may result from genetic or epigenetic modifiers influencing immune tolerance [38, 39, 40, 41]. Further studies are needed to confirm these observations and define the broader clinical spectrum of IKBKB‐related diseases.

Despite these limitations, our data expand the known spectrum of pathogenic *IKBKB* variants and highlight the mechanistic link between impaired IKKβ function and autoimmunity. Whereas bi allelic loss‐of‐function variants cause severe combined immunodeficiency (SCID) [13, 14, 36], heterozygous gain‐of‐function variants result in a spectrum of autoimmune and autoinflammatory manifestations including progressive combined immunodeficiency with declining B‐ and T‐cell numbers and function over time [15, 16, 17]. In contrast, the variant described here represents a heterozygous loss‐of‐function allele causing haploinsufficiency with diminished IKKβ activity and reduced NFKB1‐encoded p50 expression, likely reflecting its truncating nature and loss of the essential NEMO‐binding domain, unlike previously reported missense variants that preserve overall protein structure [15, 16, 17, 33].

Notably, Tuijnenburg et al. [8], reported that approximately 48% of individuals with heterozygous *NFKB1* loss‐of‐function variants exhibited autoimmune manifestations, underscoring the essential role of canonical NF‐κB signalling in immune tolerance. The parallel reduction in p50 expression observed in our patient supports a shared downstream mechanism, whereby impaired IKKβ activity disrupts NFKB1 processing and phenocopies the effects of *NFKB1* haploinsufficiency. Thus, although additional reports will be needed to establish this genotype–phenotype relationship with confidence, our findings provide strong mechanistic evidence that heterozygous *IKBKB* loss‐of‐function may drive autoimmune disease through disruption of canonical NF‐κB signalling [2, 4].

Collectively, this case illustrates the potential phenotypic variability associated with *IKBKB* variants, which have been linked to presentations ranging from severe immunodeficiency in bi‐allelic forms to immune dysregulation syndromes in heterozygous gain‐ and now, loss‐of‐function variants. Our findings suggest that partial loss of IKKβ function may not cause overt immunodeficiency, but rather tip the balance toward aberrant immune activity and autoimmunity.

## Author Contributions

Kiril Malovitski, Noy Keller Rosenthal, Eli Sprecher, Mor Pavlovsky contributed to the conceptualization of the research. Kiril Malovitski, David Hagin, Mor Pavlovsky contributed to data curation. Kiril Malovitski, Noy Keller Rosenthal, Lubna Khair, Tal Freund, Eli Sprecher, Alon Peled, Yarden Feller, Rawaa Ishtewy, Janan Mohamad, Ofer Sarig contributed to the investigation. David Hagin, Tal Freund, Rawaa Ishtewy, Alon Peled contributed to methodology and resources, Liat Samuelov, Eli Sprecher contributed to funding acquisition and project administration. Liat Samuelov, Eli Sprecher, Mor Pavlovsky contributed to the supervision, Kiril Malovitski, Mor Pavlovsky contributed to the formal analysis. Kiril Malovitski contributed to validation and visualisation. Kiril Malovitski, Mor Pavlovsky, David Hagin, Liat Samuelov, Eli Sprecher wrote the original draft of the manuscript. All authors reviewed and edited the manuscript and approved the final version for submission. Ram's family contribution was a generous philanthropic donation.

## Conflicts of Interest

The authors declare no conflicts of interest.

## Supporting information


**Figure S1:** Immune phenotyping of patient peripheral blood mononuclear cells (PBMCs).
**Figure S2:** Complementary DNA (cDNA) Sanger sequencing confirmed the expression of the aberrant transcript.
**Figure S3:** *IKBKB* mRNA expression in transfected keratinocytes.
**Figure S4:** Intracellular localization of the p.Met455fsTer1 variant.
**Figure S5:** *IKBKB* mRNA expression following *IKBKB*‐specific silencing.
**Figure S6:** IkBa degradation and P65 phosphorylation.
**Table S1:** Sequence of oligonucleotides used for *IKBKB* direct sequencing.
**Table S2:** Sequence of oligonucleotides used for RT‐qPCR.
**Table S3:** List of antibodies.
**Table S4:** List of flow‐cytometry antibodies.

## Data Availability

Full exome sequencing data are not available to protect patient privacy; anonymized variant data will be made available upon reasonable request. The authors declare that all other data are contained within the manuscript and supplemental materials.
