# Supplementary material for: Defective Function of Inhibitor of κB Kinase Subunit Beta Associated With Multiple Immune‐Mediated Disorders
Source: Exp Dermatol. 2026 Jan 14;35(1):e70206. doi: 10.1111/exd.70206 (PMC12801177; doi:10.1111/exd.70206)
Supplement: Supplementary file 1 — Figure S1: Immune phenotyping of patient peripheral blood mononuclear cells (PBMCs). Figure S2: Complementary DNA (cDNA) Sanger sequencing confirmed the expression of the aberrant transcript. Figure S3: IKBKB mRNA expression in transfected keratinocytes. Figure S4: Intracellular localization of the p.Met455fsTer1 variant. Figure S5: IKBKB mRNA expression following IKBKB‐specific silencing. Figure S6: IkBa degradation and P65 phosphorylation. Table S1: Sequence of oligonucleotides used for IKBKB direct sequencing. Table S2: Sequence of oligonucleotides used for RT‐qPCR. Table S3: List of antibodies. Table S4: List of flow‐cytometry antibodies. [file EXD-35-e70206-s001.docx]

**Supplemental information**

**Defective function of inhibitor of κB kinase subunit beta associated with multiple immune-mediated disorders**

Kiril Malovitski^1,2^, Noy Keller Rosenthal^1^, Lubna Khair^1^, David Hagin^2,3^, Tal Freund^3^, Eylon Sharoni^1^, Alon Peled^1^, Yarden Feller^1,2^, Rawaa Ishtewy^1^,Janan Mohamad^1^ ,Ofer Sarig^1^ ,Liat Samuelov^1,2^, Eli Sprecher ^1,2^, Mor Pavlovsky^1^

^1^Division of Dermatology, Tel Aviv Sourasky Medical Center, Tel Aviv, Israel; ^2^Gray Faculty of Medical & Health Sciences, Tel Aviv University, Tel Aviv, Israel; ^3^Division of Internal Medicine, Allergy and Clinical Immunology, Tel Aviv Sourasky Medical Center, Tel Aviv, Israel

**Supplementary methods**

*Cell immunofluorescence staining*

Cell immunofluorescence studies were performed with primary keratinocytes(KCs) as previously described(1). The complete list of antibodies and dilutions used can be found in **Table S3**. Staining was visualized using an LSM 700 confocal microscope (Carl Zeiss, Oberkochen, Germany).

*Flow Cytometry*

Peripheral blood mononuclear cells (PBMCs) were isolated from fresh blood samples using Ficoll density gradient centrifugation, and were stored in liquid nitrogen for later use.

For B-cell and T-cell immunophenotyping, PBMCs were thawed and stained for the relevant surface markers. For regulatory T cell immunophenotyping, cells were thawed and left overnight to recover in complete medium at 37°C and 5% CO_2._ The next day cells were fixed, permeabilized and stained for FOXP3 and Helios. For evaluation of IKBKB degradation and P65 phosphorylation, cells were thawed and stimulated with Phorbol 12-myristate 13-acetate (PMA) (15ng/mL) and Ionomycin (1M) for 15 minutes, followed by fixation, permeabilization and staining for IKBKB and pP65.

Conjugated Fluorescence-Activated Cell Sorting (FACS) antibodies, as well as fixation and permeabilization reagents used in these experiments, are listed in **Table S4**. Cells were acquired using a BD FACSCanto II flow cytometer and data analysis performed using FlowJo software (V10.0, TreeStar).

**Supplementary figures**


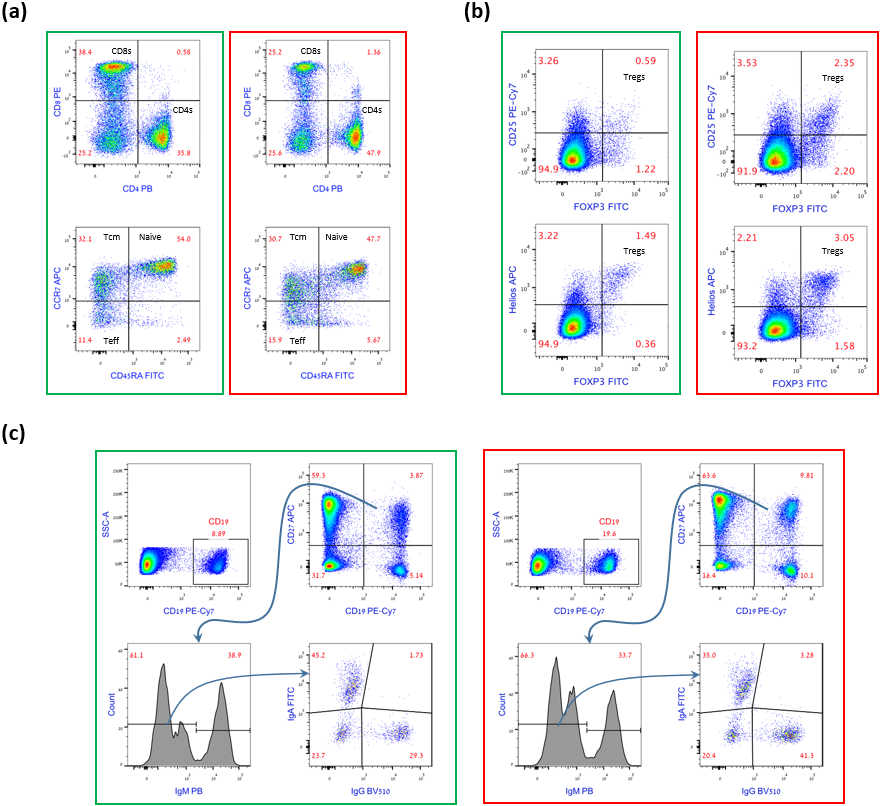


**Figure S1**

**Immune phenotyping of patient peripheral blood mononuclear cells (PBMCs).**

(a) T-cell Immunophenotyping shows normal percentages and normal ratio of CD4 / CD8 T-cells (upper panels). In addition, phenotyping showed normal T-cell maturation with normal percentages of CD45RA/CCR7 double positive Naïve cells, normal percentages of CD45RA-CCR7+ central memory T-cells (Tcm), and normal percentages of CD45RA-CCR7- Effector T-cells (Teff) (Green box- Healthy Control; Red box – Patient); (b) Treg Immunophenotyping shows normal / increased percentages of CD25highFOXP3+ Tregs (right upper quadrant, upper panels) and normal percentages of thymic derived Helios+FOXP3 Tregs (right upper quadrants, lower panels). (Left Panels; Green box- Healthy Control; Red box – Patient); (c) B-cell Immunophenotyping shows increased (~20%) percent of CD19+ B cells, with normal percent of CD27+ memory B-cells, and normal percentages of class-switched CD19+CD27+IgM-IgG/IgA+ cells. Arrows mark the gating strategy (Green box- Healthy Control; Red box – Patient).

**
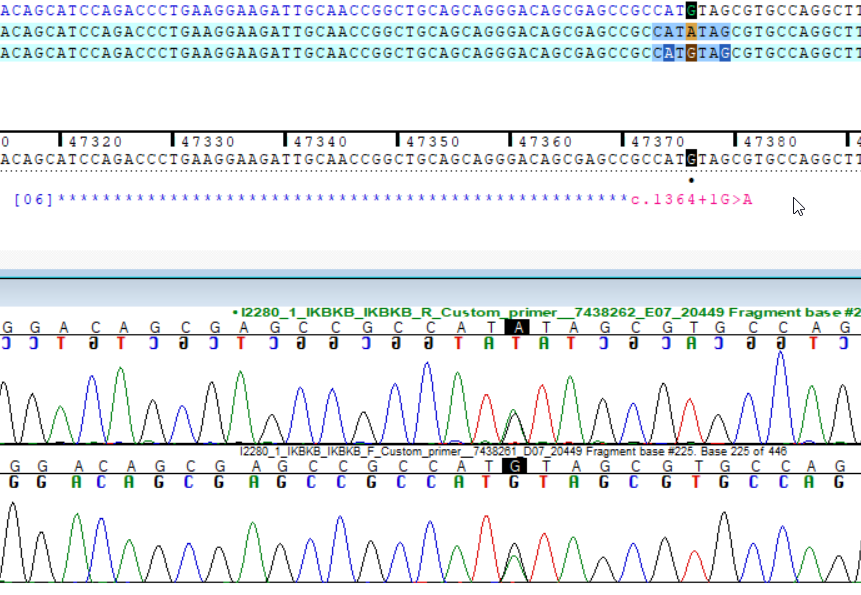
**

**
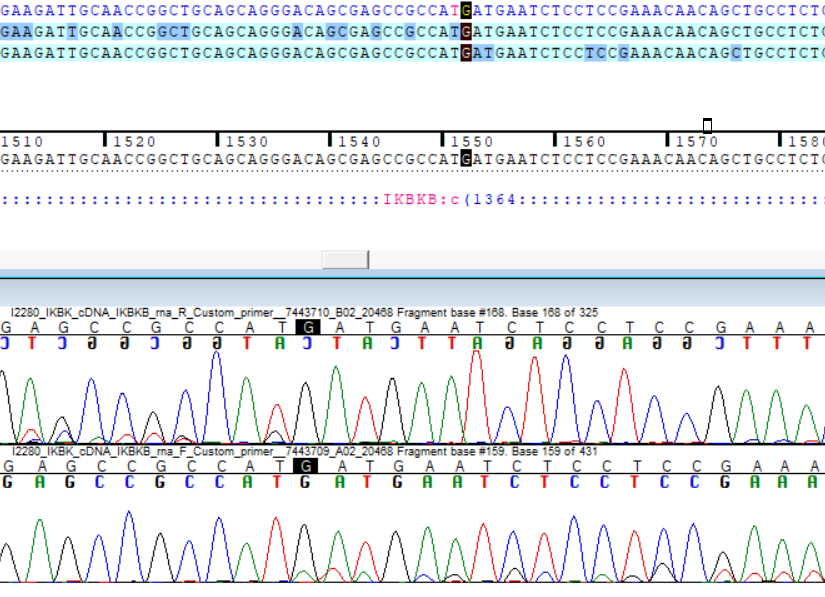
**

**Figure S2**

**Complementary DNA (cDNA) Sanger sequencing confirmed the expression of the aberrant transcript**

Sanger sequencing of genomic DNA (upper panel) and cDNA from patient-derived KCs (bottom panel). In bottom panel, high peaks correspond to the canonical transcript, while low peaks indicate the aberrant transcript. The variant site is marked by a black arrow in both panels.


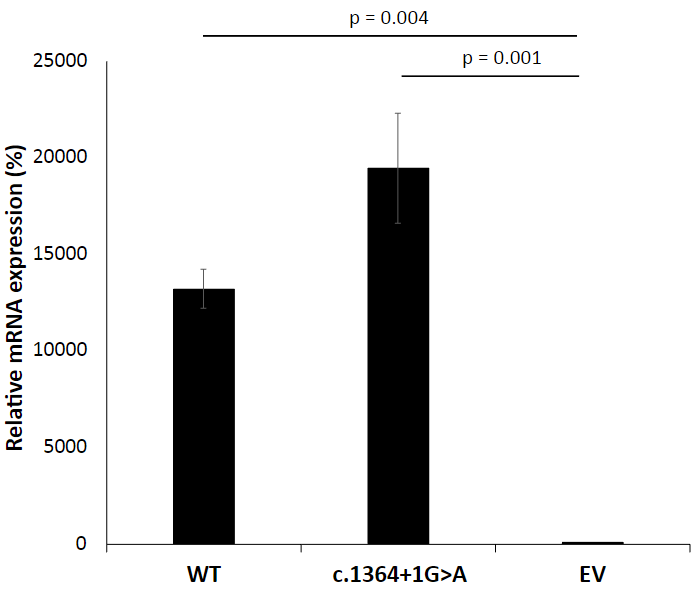


**Figure S3**

**IKBKB mRNA expression in transfected keratinocytes**

*IKBKB* mRNA levels were quantified using RT-qPCR in primary human keratinocytes (KCs) transfected with expression vectors harboring the wild-type (WT) *IKBKB* DNA sequence or *IKBKB* construct harboring the c.1364+1G>A variant. As a control, KCs were transfected with an empty vector (EV). Results were normalized to *GAPDH* mRNA, represent the mean ± SE of three experiments and are expressed as a percentage of *IKBKB* mRNA expression in cells transfected with an empty (EV) vector (p values were calculated using one-way Anova and Tukey HSD test).


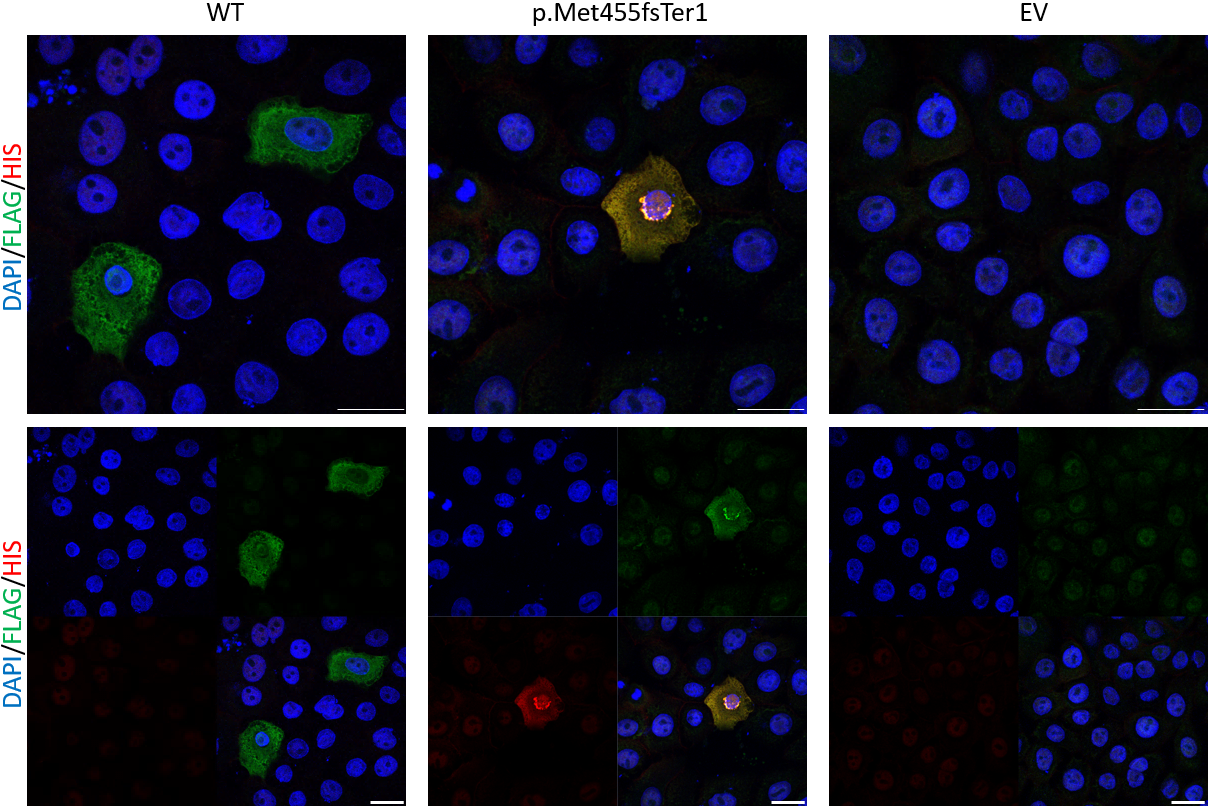


**Figure S4**

**Intracellular localization of the p.Met455fsTer1 variant**

Primary keratinocytes (KCs) were transfected with expression constructs harboring either *IKBKB* wild-type sequence (WT) or the p.Met455fsTer1 variant. As a control, KCs were transfected with an empty vector (EV). Next, the primary KCs were immunostained using a tag shared between the two constructs (green) and a tag specific for the construct harboring the p.Met455fsTer1 variant. White arrows points to aggregates in the peri-nuclear region [scale bar = 10 μm; red = 6xHIS-tagged IKBKB; green = FLAG-tagged IKBKB; blue = 4ʹ,6-diamidino-2-phenylindole (DAPI)-stained nuclei]. Split fluorescence channels corresponding to individual markers are presented in the bottom panel.


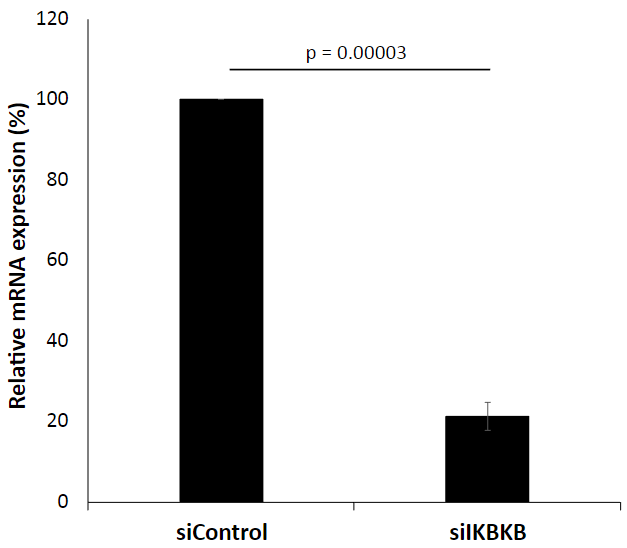


**Figure S5**

***IKBKB* mRNA expression following *IKBKB*-specific silencing**

(a) *IKBKB* mRNA levels were quantified using RT-qPCR in primary human keratinocytes transfected with *IKBKB*-specific (si*IKBKB*) or scramble (siControl) siRNAs. Results were normalized to *GAPDH* mRNA, represent the mean ± SE of three experiments and are expressed as percentage of *IKBKB* mRNA expression in cells transfected with the scramble siRNA (p values were calculated using the two-way *t*-test).


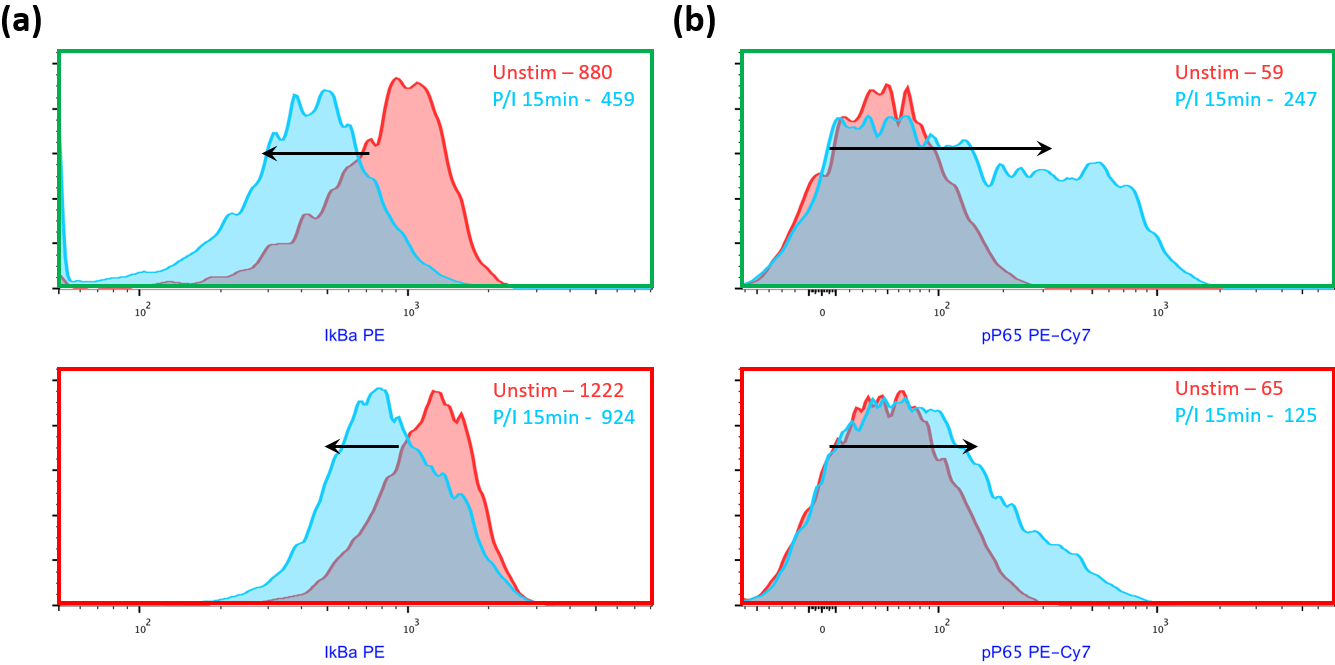


**Figure S6**

**IkBa degradation and P65 phosphorylation**

**(a)** Peripheral blood mononuclear cells (PBMCs) were stimulated with PMA and Ionomycin for 15min. Following stimulation cells were fixed, permeabilized and stained for IkBa and p65. IkBa staining (left panels) shows reduced IkBa degradation in patient’s PBMCs (lower left) compared with control (upper left); **(b)** Similarly, patient’s cells showed reduced p65 phosphorylation (lower right) compared with control cells (upper right). Numbers represent Mean Fluorescent Intensity (MFI). Cell presented are CD3 positive. (Green box- Healthy Control; Red box – Patient).

**Supplementary tables**

**Table S1. Sequence of oligonucleotides used for *IKBKB* direct sequencing**

| **Forward oligonucleotide sequence** | **Reverse oligonucleotide sequence** | **Expected product size (bp)** |
| --- | --- | --- |
| CGTGAAAGTGCCAGTAGTGTCG | TTCAGTTCCACACTCCTCACCC | 449 |

**Table S2. Sequence of oligonucleotides used for RT-qPCR**

| **Geneu** | **Forward oligonucleotide sequence** | **Reverse oligonucleotide sequence** |
| --- | --- | --- |
| ***GAPDH*** | GAGTCAACGGATTTGGTCGT | GACAAGCTTCCCGTTCTCAGCC |
| ***IKBKB*** | AGTCTTTGCACATCATTCGTGG | GTGATGCACTCAAAGGCCAGG |
| ***TNF*** | ACTTTGGAGTGATCGGCC | GCTTGAGGGTTTGCTACAAC |
| ***IL6*** | GTAGTGAGGGAACAAGCCAGAGC | TACATTTGCCGAAGAGCCCT |
| ***IL1B*** | ATGCACCTGTACGATCACTG | ACAAAGGACATGGAGAACACC |

**Table S3. List of antibodies**

| **Antigen** | **Type** | **Host** | **Dilution** | **Manufacturer** | **Catalogue number** |
| --- | --- | --- | --- | --- | --- |
| NF-κB1 p105/p50 | Monoclonal | Rabbit | 1:1000 WB | Cell Signaling | #13586 |
| DDDDK | Polyclonal | Rabbit | 1:5000 WB  1:500 IF | Abcam | ab1162 |
| 6x-His | Monoclonal | Mouse | 1:500 IF | Invitrogen | MA1-21315 |
| V5 | Monoclonal | Mouse | 1:5000 WB 1:500 IF | Invitrogen | R960-25 |
| β-Actin | Monoclonal | Mouse | 1:10000 WB | Abcam | ab8224 |
| Mouse IgG | Polyclonal | Goat | 1:10000 WB | Jackson | 115-035-003 |
| Rabbit IgG | Polyclonal | Goat | 1:5000 WB | Sigma-Aldrich | 12-348 |
| Mouse IgG | Polyclonal | Goat | 1:200 IF | Invitrogen | R-6393 |
| Rabbit IgG | Polyclonal | Goat | 1:200 IF | Invitrogen | 35553 |

WB – Western blot, IF - Immunofluorescence

**Table S4. List of flow-cytometry antibodies**

| **Antigen** | **Type** | **Host** | **Dilution** | **Manufacturer** | **Catalogue number** |
| --- | --- | --- | --- | --- | --- |
| CD19 | Monoclonal | Mouse | See product datasheet | Biolegend | 302216 |
| CD27 | Monoclonal | Mouse | See product datasheet | Biolegend | 356424 |
| IgM | Monoclonal | Mouse | See product datasheet | Biolegend | 314514 |
| IgA | Polyclonal | Goat | See product datasheet | Southren Biotech | 2050-02 |
| IgG | Monoclonal | Rat | See product datasheet | Biolegend | 410716 |
| BAFF-Receptor | Monoclonal | Mouse | See product datasheet | Biolegend | 316906 |
| CD38 | Monoclonal | Mouse | See product datasheet | Biolegend | 303522 |
| IgD | Monoclonal | Mouse | See product datasheet | Biolegend | 348220 |
| CD10 | Monoclonal | Mouse | See product datasheet | Biolegend | 312212 |
| CD24 | Monoclonal | Mouse | See product datasheet | Biolegend | 311106 |
| CD21 | Monoclonal | Mouse | See product datasheet | Invitrogen | 62-0219-42 |
| CD4 | Monoclonal | Mouse | See product datasheet | Biolegend | 300521 |
| CD25 | Monoclonal | Mouse | See product datasheet | Biolegend | 302612 |
| CD127 | Monoclonal | Mouse | See product datasheet | Biolegend | 351304 |
| FOXP3 | Monoclonal | Mouse | See product datasheet | Biolegend | 320212 |
| Helios | Monoclonal | Mouse | See product datasheet | Biolegend | 137218 |
| CTLA4 | Monoclonal | Mouse | See product datasheet | Biolegend | 369608 |
| CD8a | Monoclonal | Mouse | See product datasheet | Biolegend | 301008 |
| CD45RA | Monoclonal | Mouse | See product datasheet | Biolegend | 304106 |
| CD45RO | Monoclonal | Mouse | See product datasheet | Biolegend | 304228 |
| HLA-DR | Monoclonal | Mouse | See product datasheet | Biolegend | 307616 |
| CCR7 | Monoclonal | Mouse | See product datasheet | Biolegend | 353218 |
| CD3 | Monoclonal | Mouse | See product datasheet | Biolegend | 317306 |
| IkBa | Monoclonal | Rat | See product datasheet | Invitrogen | 12-9036-42 |
| Phospho-P65 | Monoclonal | Mouse | See product datasheet | BD Biosciences | 560335 |
| Phosphor-S6 | Monoclonal | Human cell line | See product datasheet | Miltenyi Biotec | 130-124-255 |

1. Mohamad J, Sarig O, Beattie P, Malovitski K, Assaf S, O'Toole E, et al. A unique skin phenotype resulting from a large heterozygous deletion spanning six keratin genes. Br J Dermatol. 2022;187(5):773-7.
